# A System of Clinical Medicine

**Published:** 1848-10

**Authors:** 


*A System of Clinical Medicine.* By Robert James Graves, M. D., M. R. I. A., one of the Physicians of the Meath Hospital and County of Dublin Infirmary; formerly Professor of the Institutes of Medicine, etc., etc. *With Notes and a series of Lectures, by* W. W. Gerhard, M. D., Lecturer on Clinical Medicine to the University of Pennsylvania; one of the Physicians to the Pennsylvania Hospital, etc., etc. *Third American Edition.* 8vo. pp. 751. Edw. Barrington & Geo. D. Haswell. Philadelphia: 1848.

The Clinical Lectures of Dr. Graves have long had a deservedly high reputation. With a large share of enthusiasm, which gives warmth and interest to his descriptions of phenomena, we are continually struck with the boldness and originality of views presented by the author for our consideration, and one can hardly follow him without being strongly impressed with the conviction that the writer has examined his subject carefully and understandingly.

We have long been impressed with the similarity of views and modes of practice which obtain in the Irish metropolis and in Philadelphia. Indeed, we have repeatedly heard the observation made by gentlemen direct from the schools and hospitals in Dublin, while listening to the lectures and witnessing the treatment of patients in our own. Some differences, particularly in the phenomena which are presented during epidemics, proceeding from climatic or other local influences, may be observed in their descriptions of disease, and some slight differences of opinion as to the efficacy of particular remedies, owing, partly to the modifications mentioned, and partly, perhaps, to the force of habit, or other causes operating upon individual minds; but, as a general rule, the descriptions of individual diseases as observed in the one city, and the treatment recommended by eminent practitioners, will be found to be very nearly the same in the other. Hence it is, that the writings of Colles, of Crampton, of Graves, and Stokes, and Churchill, are so well regarded among us.

"Since the last edition of Dr. Graves's Lectures was published in this city," says the Editor, "a new one, considerably enlarged, has been published by Dr. Graves, in Europe: from this edition the present one is printed. It contains the whole of the Lectures, which make a very large volume; but some detached papers, which were not printed in the former editions, and were first published in the present one, (English edition?) are not reprinted. These essays the publishers will probably issue in a separate form. The demand for a new edition is the best proof of the high character these Lectures have gained in the United States, and of the very extended reputation they have justly attained.

"The additional lectures, (by the editor,) which at the request of the publishers, were appended to the last edition of the work, have been revised, and some new matter on the subject of Typhus Fever has been added."

